# Prevalence of Oral Potentially Malignant Lesions, Tobacco use, and Effect of Cessation Strategies among Solid Waste Management workers in Northern India: a pre-post intervention study

**DOI:** 10.1186/s12903-024-05087-8

**Published:** 2024-10-26

**Authors:** Divya Khanna, Tulika Shruti, Manish Tiwari, Priyanka Sharma, Aqusa Khan, Sudhir Ranjan, P. Balasundaram, Naveen Khargekar, Pankaj Chaturvedi, Aseem Mishra

**Affiliations:** 1 Department of Preventive Oncology, Homi Bhabha Cancer Hospital (HBCH) and Mahamana Pandit Madan Mohan Malaviya Cancer Centre (MPMMCC), Tata Memorial Centres, Varanasi, Uttar Pradesh 221005 India; 2 Homi Bhabha National Institute (HBNI), Mumbai, 400094 India; 3 Head and Neck Surgical Oncology, Karkinos Healthcare, Kolkata, India; 4 Dept of Community Medicine, Vardhman Mahavir Medical College and Safdarjung Hospital, New Delhi, 110029 India; 5 National Institute of Immunohematology, Indian Council of Medical Research, Mumbai, 400012 India; 6 Tata Memorial Hospital, Mumbai, 400012 India; 7 Advanced Centre for Treatment Research and Education in Cancer (ACTREC), Tata Memorial Centre, Mumbai, 410210 India; 8 Department of Head and Neck Surgical Oncology, Homi Bhabha Cancer Hospital (HBCH) and Mahamana Pandit Madan Mohan Malaviya Cancer Centre (MPMMCC), Tata Memorial Centres, Varanasi, Uttar Pradesh 221005 India

**Keywords:** Oral cancer, Smokeless tobacco, Occupational health, Tobacco cessation, Nicotine dependence, Behavioral interventions

## Abstract

**Background:**

India bears the highest global burden of oral cancer, despite having an operational tobacco cessation framework. Occupational groups like solid waste management personnel face significant health challenges due to prevalent tobacco use, leading to oral potentially malignant lesions and oral cancer. Enhanced tobacco control strategies are essential for these groups.

**Methods:**

A pre-post interventional, community-based study enrolled 1200 municipal workers in Varanasi, India, from July 2022 to August 2023. 858 tobacco users underwent screening and were randomly assigned to one of three interventions: Very Brief Advice, Individual Behavioral Counseling, or Group Behavioral Therapy. Follow-up was conducted one year after the baseline interventions Effectiveness was measured by nicotine dependence reduction using the Fagerstrom Test for Nicotine Dependence (FTND) scores and cessation rates defined as at least 6-month abstinence. Appropriate statistical tests assessed the burden of tobacco use, oral potentially malignant lesions, and pre-post differences in FTND scores within and between groups. A p-value of < 0.05 was considered statistically significant.

**Results:**

Municipal workers exhibited a high prevalence (71.5%) of smokeless tobacco (SLT) use. One-third (32.9%) of the participants screened positive for oral potentially malignant lesions and oral cancer. Leukoplakia was the most common lesion. Screened positivity correlated with significant nicotine dependence. Among 494 follow-up participants, 47.1% reported a significant reduction in nicotine dependence across all intervention groups. Quade’s ANCOVA indicated significant differences in post-test FTND scores, with individual behavioral counselling showing the greatest reduction. However, no cessation was achieved in any group despite the significant decline in dependence.

**Conclusion:**

Solid waste management personnel in Varanasi show heightened SLT use and associated oral potentially malignant lesions. The persistent tobacco use in these high-risk occupational populations undermines government tobacco control efforts and highlights the need for robust policy and implementation strategies. The study demonstrated a significant reduction in nicotine dependence following interventions, though tobacco cessation was not achieved. More frequent interventions and addressing quitting barriers—such as cultural norms, lack of awareness, easy accessibility, and adverse working conditions—are crucial. Developing a tailored workplace model to tackle tobacco use in occupational settings may facilitate cessation.

**Clinical trial registration number:**

Trial registration Clinical Trials Registry India CTRI/2020/07/026479. Date of registration 10/07/2020.

**Supplementary Information:**

The online version contains supplementary material available at 10.1186/s12903-024-05087-8.

## Introduction

According to the World Health Organization (WHO), 80% of the world’s 1.3 billion tobacco users live in low- and middle-income countries (LMICs) [1]. The majority (90%) of smokeless tobacco (SLT) consumers reside in Southeast Asia [2]. About 20 million Indian adults regularly consume some form of SLT (21.4% overall, 29.6% men, and 12.8% women), which is twice the prevalence of smoking [3]. SLT is closely associated with the development of oral potentially malignant lesions (OPML) such as leukoplakia, erythroplakia and oral sub-mucous fibrosis [4]. OPMLs exhibit variability in their progression malignant transformation rate of 1.4-49.5% over a follow-up period ranging from 12 months to 20 years [5]. Furthermore, misconceptions surrounding tobacco use further perpetuate its prevalence. Many individuals erroneously believe that tobacco aids in concentration, suppression of appetite, anxiety reduction, induces relaxation, and provides pleasure [6]. These misconceptions contribute to the continued use of tobacco, particularly among daily wage workers and individuals of low socioeconomic status [7, 8].

Oral Cancer (OC) presents a significant challenge in the Indian subcontinent, ranking among the top three sites of cancer in the country [9]. OC in India accounts for over 30% of all cancers. Furthermore, the majority of the OCs are diagnosed in advanced stages and have poor prognosis and survival [10]. Population-based cancer registry (PBCR) serves as an essential tool for determining the cancer burden at both state and national levels in India and is recognized as a crucial component of national cancer control programs [11]. PBCR of Varanasi (a district in the Uttar Pradesh state of India) has recorded the second-highest burden of OC across all Indian PBCRs [11]. Notably, one in 15 males and one in 17 females in this district are at risk of developing OC [11]. This is attributed mainly to the prevalent use of tobacco, mainly as SLT with or without the use of areca nut in this region [11].

A recent systematic review and meta-analysis of 19 studies with 24,498 participants worldwide demonstrated the effectiveness of behavioral interventions for SLT cessation in adults (relative risk {RR} of 1.63, 95% Confidence Interval {CI}: 1.32–1.94), showing efficacy in both developed (RR = 1.39, 95% CI: 1.16–1.63) and developing countries (RR = 2.79, 95% CI: 2.32–3.25) [12]. Similarly, a prior meta-analysis of 16 Indian studies involving 9,613 participants highlighted the overall efficacy of non-pharmacological behavioral interventions for tobacco cessation among any form of tobacco users, with a pooled RR of 1.73 (95% CI: 1.58–1.90) using a fixed-effect model, and 2.02 (95% CI: 1.64–2.48) using a random-effects model. Additionally, behavioral interventions targeting smoking and SLT users proved effective, with RRs of 1.69 (95% CI: 1.50–1.90) and 2.12 (95% CI: 1.49–3.01) [13].

Indian waste management personnel (also called Municipal or Nagar Nigam Workers) undertake critical tasks such as garbage collection, sorting recyclable materials, and waste disposal, which expose them to heightened occupational health hazards [14]. It is essential to recognize that these dedicated workers predominantly hail from low socioeconomic backgrounds [15]. According to a systematic review, individuals belonging to low socioeconomic classes face a significantly higher risk of OC compared to their counterparts in higher social strata, with an odds ratio (OR) of 1.84 and a 95% CI of 1.47–2.31 [16]. The Municipal workers frequently turn to substance use during their working hours to cope with the difficult and unsanitary conditions they encounter in their daily tasks [15]. This underscores the vulnerability of municipal workers to the adverse health effects associated with their occupation.

Previous studies conducted in India have highlighted the widespread use of tobacco among municipal workers [15, 17]. However, despite the evident health risks and the prevalence of tobacco use in this profession, there remains a notable gap in research regarding effective tobacco cessation interventions tailored specifically to address the needs of these occupational workers. Recognizing the socio-economic challenges they face; it becomes imperative to develop targeted and culturally sensitive strategies to support tobacco cessation efforts among this vulnerable population.

With this background, our study aimed to address this research gap by comprehensively assessing municipal workers in the Varanasi district of Northern India. We focused on estimating the prevalence of tobacco use, nicotine dependence and OPML observed during screening. Furthermore, we seek to evaluate the effectiveness of various tobacco cessation interventions, with a particular emphasis on community-based approaches that are attuned to the unique socio-economic context and occupational challenges these workers face.

## Methods

### Study settings

The total area under Varanasi Nagar Nigam (Municipal Corporation) is 82.10 sq. Kms with an estimated population of 1,198,491 persons (635,140 males and 563,351 females, Census 2011) [11]. There are 5 zones and 14 subzones under the Municipal Corporation constituting 3500 workers. A food and sanitary inspector heads each subzone. Due to the nature of their occupation and restrictions on smoking during working hours, workers self-reported resorting to the use of SLT. The study was undertaken by the Surgical and Preventive Oncology divisions of a government-aided tertiary cancer centre in Varanasi, as a part of a community outreach programme for cancer prevention and control. The study was held at the municipal corporation’s main office, where participants were invited to attend screening camps.

### Study design

The present study employed a pre-post intervention design to evaluate the implementation of tobacco cessation strategies among waste management personnel in India between July 2022 and August 2023. Additionally, early detection of OPML was conducted through cross-sectional screening camps. The reporting of this study adheres to the applicable guidelines from the Consolidated Standards of Reporting Trials (CONSORT) checklist extension and has followed the Template for Intervention Description and Replication (TIDieR) checklist for intervention reporting [18, 19]. (Additional files)

### Eligibility criteria

The study included municipal workers aged 18 to 60 years who consented to participation. We excluded individuals with a history of previously diagnosed OC, any acute or debilitating conditions, and those not willing to participate. Among the eligible participants, we identified the high-risk population for OPML and OC based on any of the following criteria: (i) individuals aged 18 years and above with a history of tobacco use, (ii) individuals with clinical signs and symptoms such as non-healing ulcer, white/red patch in mouth, restricted mouth opening, or neck swelling, (ii) and all individual 40 years and above irrespective of their habit status [20–23]. This high-risk population underwent OC screening.

### Ethics approval and consent to participate

#### Ethical approval

was obtained from the Institutional Ethics Committee, Mahamana Pandit Madan Mohan Malaviya Cancer Centre and Homi Bhabha Cancer Hospital, Tata Memorial Centre, Varanasi (OIEC/11000025/2021/00003), and written informed consent was secured from all willing participants, with confidentiality ensured throughout the study. Administrative approval was taken from the municipal office of the Varanasi district. The study adhered to the guidelines outlined in the Declaration of Helsinki concerning research involving human subjects.

#### Sample size and sampling frame

A total of 3500 municipal workers currently serves in the Municipality of Varanasi. The study’s sample size was determined based on several considerations. As one of the primary outcomes of our study was to assess any significant difference in nicotine dependence across the three intervention groups (very brief advice, individual behavioral counselling, group behavior therapy) we needed to calculate the effect size for repeated measures, which is commonly used in pre-post designs with more than two groups. The formula incorporates considerations for within-subject correlations and multiple groups [24, 25].

$${\bf{n}} = {\text{ }}{\left( {{\bf{Z\alpha }} + {\bf{Z\beta }}} \right)^{\bf{2}}} \times {\text{ }}{\bf{2}} \times \left( {{\bf{1}} - {\bf{\rho }}} \right)/{\text{ }}{{\bf{f}}^{\bf{2}}}$$

Following were our considerations for the sample size calculation:

##### Number of groups (k)

3 interventional groups.

##### Number of measurements (m)

As it was pre-post design, m = 2.

##### Effect size (f)

The expected effect size can be small (0.1), medium (0.25), or large (0.4) as per Cohen’s conventions. We assumed a medium effect size of 0.25. As the outcome parameter was the FTND score which is a nonparametric outcome, an asymptotic relative efficiency (ARE) correction of 0.71625 was applied to the effect size. Therefore, the corrected effect size was 0.179 which was rounded to 0.20 [26]. 

**The significance level (α)** was kept at 0.05, therefore Zα = 1.96.

**Power (1-β)** was kept at 0.80, therefore Zβ = 0.84.

**The correlation between Repeated Measures (ρ)** was assumed as 0.5.

**The Sphericity Correction Factor (ε)** was assumed to be 1 (as no specific information was available; Greenhouse-Geisser correction).

Applying these considerations to the above formula, *n* = 196 participants per group were needed to detect an effect size of 0.2 with a significance level of 0.05, a power of 0.80, and a correlation between repeated measures of 0.5.

For the intervention analysis, we determined that a total of 588 participants were needed. Assuming that 50% of the participants would be available for post-intervention evaluation after 1-year, we aimed to enroll an additional 294 participants, bringing the total to 882 participants. Additionally, based on previous studies on solid waste management workers, which reported a 77% prevalence of tobacco use [15], we expected that approximately 30% of the enrolled participants through screening camps would be non-users. To account for this proportion, we added 265 participants. This calculation resulted in a total required sample size of 1147 participants. To ensure adequate power and account for potential dropouts and non-users among the study population, we rounded this number up to 1200 participants.

A line listing of 14 subzones was created. Based on the population of each subzone, we scheduled screening and intervention camps per subzone on separate dates. These dates were randomly assigned to one of the three intervention strategies using the lottery method. Consequently, eligible workers from each subzone were randomly allocated to one of the three intervention groups.

### Methodology and data collection

#### Oral cancer screening

After obtaining written consent, the trained doctors collected socio-demographic variables such as age, gender, SLT use characteristics, and the Fagerstorm scale for SLT on a pre-defined proforma. Following this, OC screening was conducted through oral visual examination using a white flashlight. During the inspection, the labial and buccal mucosa, retromolar area, gingiva, anterior tongue, floor of the mouth, and hard palate were thoroughly examined, with palpation performed as necessary. Findings were classified into three categories: benign lesions (e.g., fissures in the tongue, aphthous ulcers, black patches, blanching), OPML (leukoplakia, erythroplakia, oral submucous fibrosis, lichen planus) [22], and suspicious lesions suggestive of cancer (e.g. ulcers or growths). Screen positivity was defined as the presence of one or more OPMLs or suspicious lesions. All screen-positive individuals were referred to the Tertiary Cancer Centre (TCC) on the same day for further diagnostic testing, which was provided free of cost. While these screen-positive individuals were recommended to visit the TCC within three months, no strict upper time limit was imposed. Patients were treated by oncologists at the TCC, following guidelines from the National Cancer Grid, and the Oral Cancer Task Force [23, 27]. Screen-negative participants were advised to undergo repeat screening after three years as per the government of India (GOI) operational guidelines [28]. 

#### Cessation interventions

A pre-post design was adopted to evaluate the effectiveness of different tobacco cessation strategies among municipal workers. Following screening, the study participants were randomly allocated to one of the three intervention groups: Very Brief Advice, Individual Behavioral Counseling, and Group Behavioral Therapy (called active interventions).

**Fig. 1:**
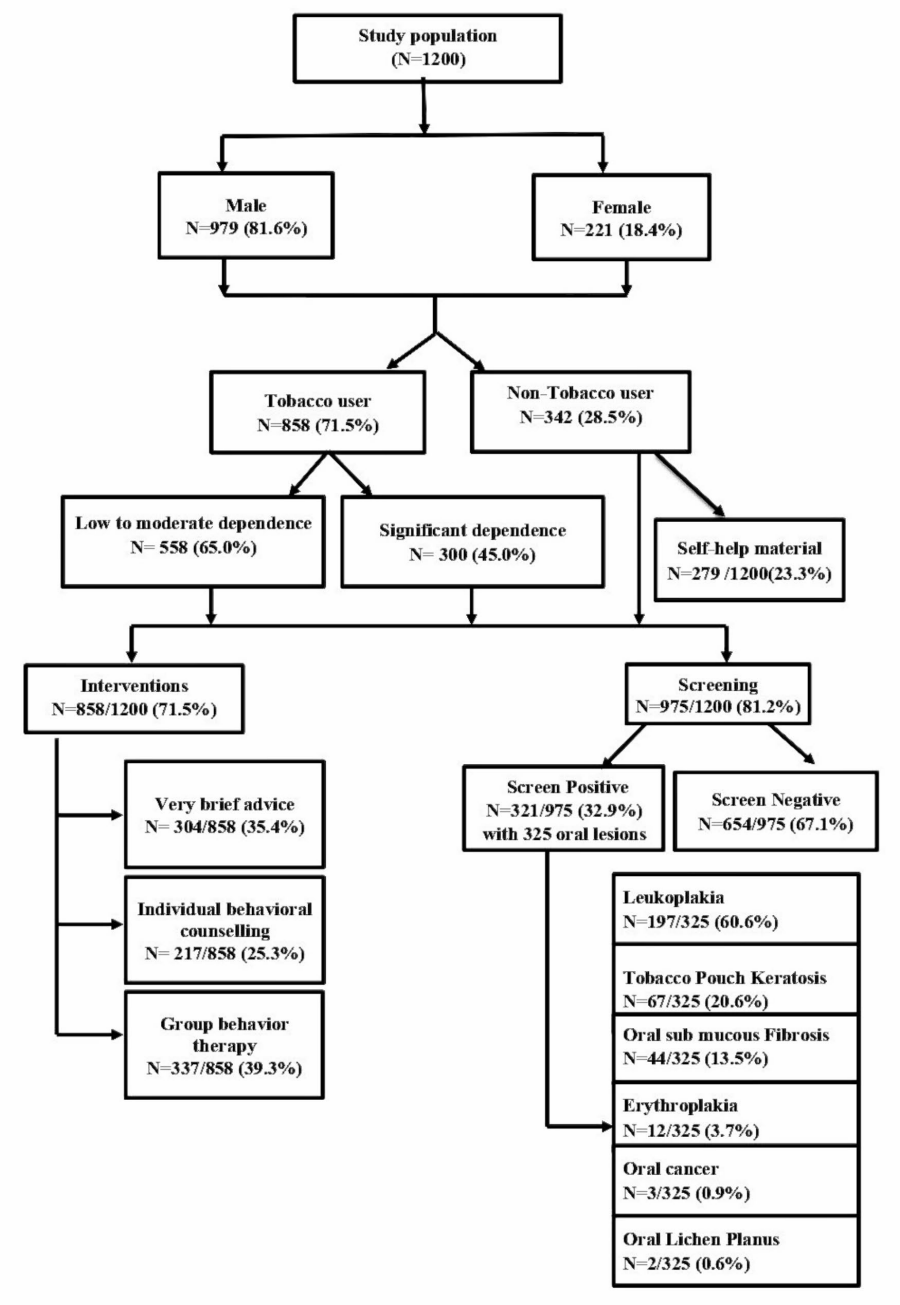
Study flow

1) **Very Brief Advice (VBA)**: A patient-centered less than 60-second counselling for health hazards of tobacco use and benefits of quitting tobacco by healthcare personnel [29]. 

2) **Individual Behavioral Counselling (IBC)**: Also called Brief Intervention (BI). This was a face-to-face interview conducted by trained doctors. This involved behavioral change techniques, and motivational interviewing to enhance the individual’s impetus to change their behavior. Behavioral cognitive therapy was given involving 5 “A” and 5 “R” techniques. This intervention lasted up to 3–5 min [30]. 

3) **Group Behavior Therapy (GBT)**: Behavioral intervention was offered to small groups of 5–10 individuals at a time, which allowed individuals to learn behavioral techniques and provide peer support. This intervention lasted 5–10 min [31] (Fig. 1).

The OC screening was conducted by head and neck oncology surgeons following standardized IARC guidelines, ensuring consistent screening outcomes. Behavioral interventions were administered by preventive oncology doctors using standardized WHO [30] and National Tobacco Quitline [32] protocols for tobacco cessation, minimizing variability. Demographic and habit-specific data were recorded by a trained field investigator using the pilot-tested proforma.

As this was a high-risk occupational study population, it would have been ethical to provide some form of risk factor prevention to the never-users. Hence, we offered self-help health education material to all never-users as a risk factor prevention strategy. The self-help material was printed in the local language regarding the health hazards of tobacco use, the benefits of quitting tobacco and early detection of OC. Participants underwent follow-up after 1 year to evaluate their efforts to quit tobacco by evaluating their nicotine dependence and cessation rates. All participants received tobacco cessation advice in their follow-up visit.

### Study outcomes

The effectiveness of tobacco cessation strategies was evaluated through changes in nicotine dependence using the validated tool of Fagerstrom Nicotine Dependence Scale-Smokeless tobacco (FTND-ST) [33] at the baseline and the follow-up point. Vidyasagaran et al. translated and validated the FTND tool in the Hindi language through standard guidelines. A rigorous translation process was followed, involving bilingual translators for forward and back-translation. A joint committee reviewed and resolved discrepancies, ensuring cultural and linguistic accuracy. Pre-testing led to minor revisions without altering scoring or scales. (Supplementary material) [34] A score of 4 or less was classified as indicating low to moderate dependence, whereas a score of 5 or higher was categorized as significant dependence [33]. Tobacco use cessation rates were defined as at least a 6-month continuous abstinence from SLT use. Screening outcomes were noted as OPML and OC based on the operational definitions of the IARC training manual [22]. Screening socio-demographic information such as the age and gender of the participants were collected on a pre-defined proforma.

### Data analysis

The data collected were entered into Microsoft Excel Sheet (version 2007) and analyzed using the Statistical Package for Social Sciences (SPSS version 22.0). Descriptive statistics, such as mean (standard deviation {SD}), and percentages were calculated. To compare the proportion differences between categorical variables, the Chi-squared or Fisher’s exact test was employed. We assessed nicotine dependence with study variables, including gender and age. Additionally, we examined the association between screening outcomes and factors such as gender, age, and nicotine dependence. The changes in FTND (Fagerstrom Test for Nicotine Dependence) scores from pre- to post-intervention were calculated within and between the three active intervention groups. These changes were reported as the mean change in nicotine dependence among ever tobacco users.

We avoided traditional parametric tests because the pre-and post-test FTND mean scores across the three groups were not normally distributed and differed significantly at the pre-test level. Additionally, FTND scores are ordinal, not interval, violating parametric test assumptions. Consequently, we used non-parametric tests to assess differences in mean scores within and between groups.

(i) Within-Group Comparisons: We used the Wilcoxon signed-rank test to assess significant differences in pre- and post-test mean scores within each group.
(ii) Between-Group Comparisons: To test for significant differences in pre-and post-test scores between the three intervention groups, we used a non-parametric variant of ANCOVA, namely Quade’s ANCOVA, to generate an F statistic [35]. A post-hoc analysis using Scheffé’s assumption for equal variances was conducted to explore any significant differences in the post-test scores between the three intervention groups. A *p <* 0.05 was considered statistically significant.

## Results

During the study period, a total of 60 camps were conducted to enroll the targeted 1200 eligible participants. The study population characterized a predominantly male representation, comprising 81.6% (979), while females constituted 18.4% (221) of the sample. Among these participants, 71.5% (858) were identified as tobacco users and all of them were current users of SLT, while the remaining 28.5% (342) were non-users. Levels of dependence within the tobacco users revealed that 65.0% (558) had low to moderate dependence, and 35.0% (300) exhibited significant dependence. There was no significant difference in the distribution of the level of nicotine dependence, gender, or age (Table 1).

Active interventions were given to the 858 tobacco users in the study population. Specific tobacco cessation interventions such as very brief advice (35.4%, 304/858), individual behavioral counselling (25.4%, 217/858), and group behavior therapy (39.3%, 337/858) were allocated (Fig. 1). The never users were provided with self-help educational material for risk factor prevention (Fig. 1).

We screened 975 high-risk study participants (tobacco users as well as non-users aged 40 years and above). Among them, 321 (32.9%) participants were screened positive for 325 OPML and OC. Among the screened positive, leukoplakia emerged as the most prevalent OPML (60.6%, 197/325), followed by tobacco pouch keratosis (20.6%, 67/325), oral submucous fibrosis (13.5%, 44/325), erythroplakia (3.7%, 12/325), oral cancer (0.9%, 3/325), and oral lichen planus (0.6%, 2/325). (Figure-1) There was no significant association between the screening outcomes and the gender or age of the screened population. However, significant nicotine dependence was associated with higher screened positivity (Table 2). Additionally, lesions such as erythroplakia and those suspicious for cancer were significantly more common in participants with high tobacco dependence, while leukoplakia, OSMF, and tobacco pouch keratosis were more frequently observed in those with low to moderate dependence (Table 3).

**Fig. 2:**
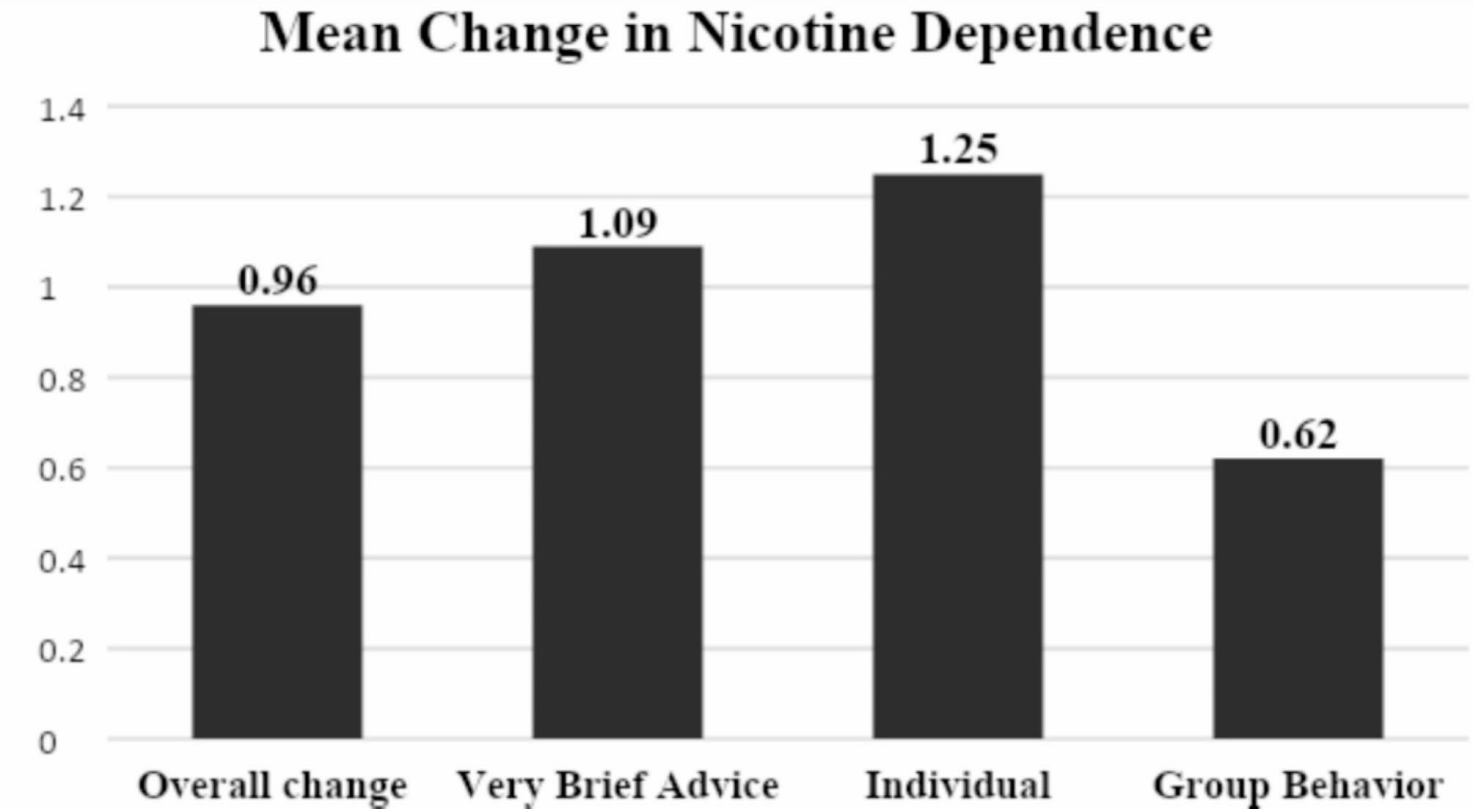
Pre-post changes in mean scores of the Fagerstrom Test for Nicotine Dependence from baseline to follow-up in the different intervention groups

At the end of one year, among the 858 tobacco users who received one of the active interventions, 494 (57.5%) could be followed to assess the impact of various interventions. Overall, 233 out of 494 (47.1%) followed-up participants reported reduced nicotine dependence. Out of which 25 (10.7%) reported no nicotine dependence on follow-up. Figure 2. displays the mean change in FTND score from baseline to follow-up within the intervention groups, which denoted the decline in nicotine dependence among the different active interventions. There was an overall decline in nicotine dependence among all tobacco users, regardless of the allocated intervention group. The FTND mean score significantly decreased from 3.65 (SD 2.54) at baseline to 2.69 (SD 2.21) at follow-up, with a mean difference of 0.96 (*p* < 0.001). Among the three intervention groups, IBC exhibited the highest decline in nicotine dependence (FTND mean score 2.88 (SD 1.63) at baseline to 1.63 (SD 1.48) at follow-up with a mean difference of 1.25 (*p* < 0.001), followed by VBA (FTND mean score 4.21 (SD 2.69) at baseline to 3.12 (SD 2.22) at follow-up with a mean difference of 1.09 (*p* < 0.001) and GBT (FTND score 3.56 (SD 2.52) at baseline to 2.94 (SD 2.39) at follow-up with a mean difference of 0.62 (*p* < 0.001).

We used Quade’s ANCOVA to evaluate the effectiveness of three interventions on reducing nicotine dependence, as measured by changes in post-test mean FTND scores while adjusting for baseline FTND scores. The analysis produced an F-statistic of 22.913 with an associated (*p* < 0.001). This indicates a statistically significant difference in the adjusted mean changes in FTND scores across the three intervention groups (*p* < 0.05). The post hoc analysis revealed that the post-test FTND scores for the IBC group were significantly different from those in the VBA and GBT groups, reflecting a greater reduction in post-test scores. There was no significant difference in the post-test FTND scores between the VBA and GBT groups, reflecting almost equal effectiveness for these two interventions. Though a significant decline in nicotine dependence was observed, none of the participants in the active interventions reported tobacco cessation as per the operational definition of 6 months of tobacco abstinence.

**Table 1:** Association of nicotine dependence with gender and age of the participants (*N* = 858)

Variables	Low to moderate dependence (*n* = 558) N(%)	Significant dependence (*n* = 300) N (%)	*p* value
Male	472 (63.8%)	268 (36.2%)	0.054
Female	86 (72.9%)	32 (27.1%)	
18–29 years	139 (64.6%)	76 (35.3%)	0.987
30–49 years	334 (65.2%)	178 (34.8%)	
50 years and above	85 (64.9%)	46 (35.1%)	

**Table 2:** Association of screening outcomes with age, gender and nicotine dependence

Variables	Screen Positive *N* (%)	Screen Negative *N* (%)	*p* value
Age (*n* = 975)			
18–29 years	66(30.7%)	149(69.3%)	0.501
30–49 years	203(38.7%)	388(61.3%)	
50 years and above	52(38.9%)	117(61.1%)	
Gender (*n* = 975)			
Male	273(36.2%)	538(63.8%)	0.274
Female	48(39.8%)	116(60.2%)	
Level of dependence (*n* = 858)			
Low to moderate dependence	177(31.7%)	381(68.3%)	< 0.001*
Significant dependence	138(46.0%)	162(54.0%)	

*Statistically significant

**Table 3:** Association between Tobacco Dependence with Screening outcomes (*n* = 315)

Tobacco Dependence*	Erythroplakia *N* (%)	Leukoplakia *N* (%)	Lichen Planus *N* (%)	Malignancy *N* (%)	Oral Submucous Fibrosis *N* (%)	Tobacco Pouch Keratosis *N* (%)	Fisher’s Exact test	*p* value
**Low to Moderate Dependence**	1 (0.56%)	116 (65.00%)	0 (0.00%)	0 (0.00%)	23 (12.95%)	37 (20.88%)	F test = 17.045	0.002
**Significant Dependence**	11 (7.97%)	80 (57.97%)	1 (0.72%)	3 (2.17%)	15 (10.87%)	28 (20.29%)		

*Assessed using the Fagerstorm Nicotine Dependence Test for Smokeless Tobacco

**Fig. 3:**
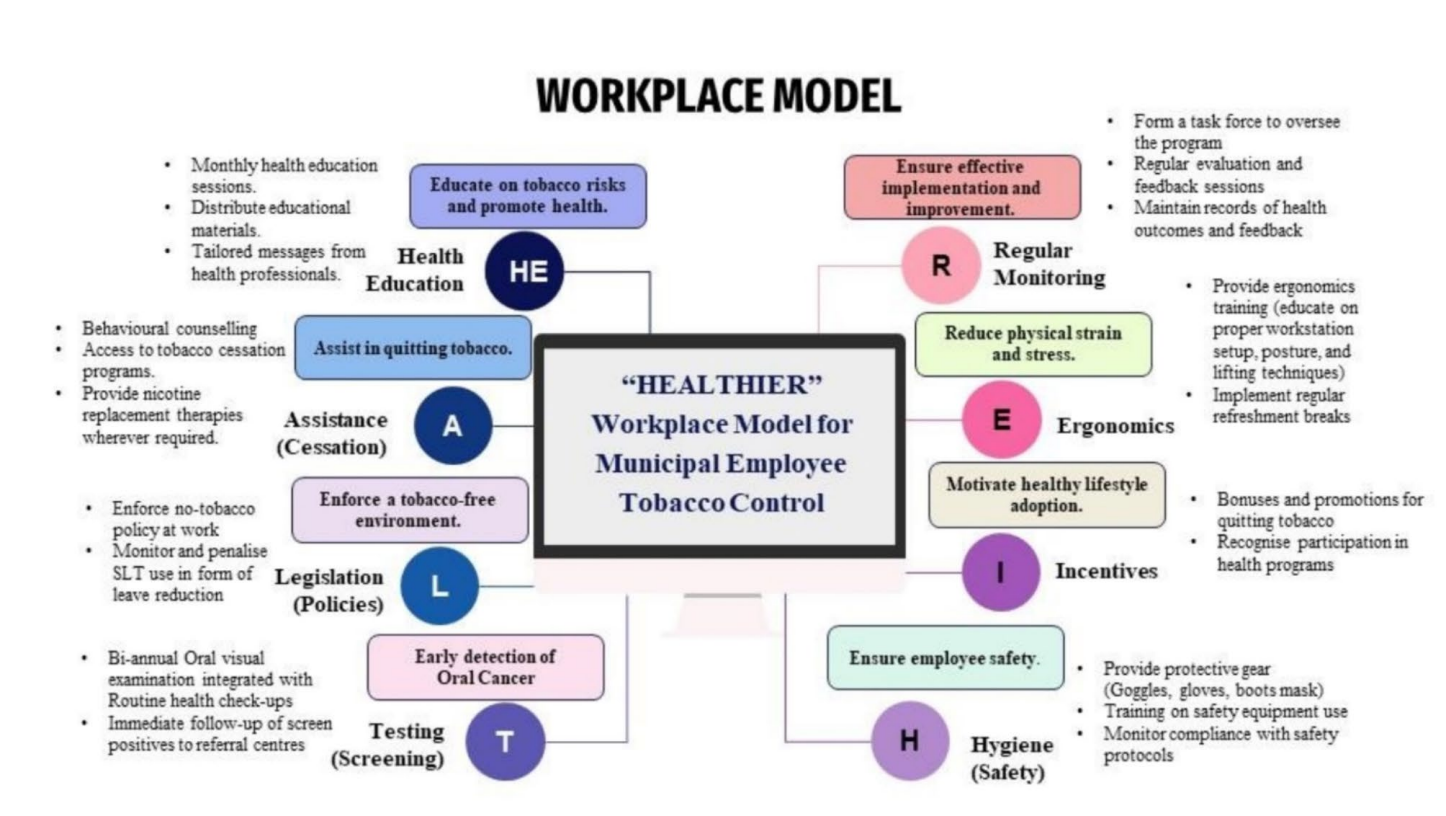
Workplace model for tobacco cessation

## Discussion

As per GLOBOCAN 2022 estimates, India has the highest burden of lip, and oral cavity cancer incidence (36.9%; 143759/ 389846) and mortality (42.4%; 79979/188438) which is attributed to the prevalent use of tobacco, especially SLT with or without areca nut [36]. Previous Indian studies have reported a much higher use of tobacco among certain occupational groups when compared to the general population [6, 8, 15, 37, 38]. In our study setting, the municipal workers are one such group with a high SLT prevalence. Hence it was imperative to conduct a study to understand the prevalence of tobacco use, dependence, OPML, and the effectiveness of the different tobacco cessation interventions in this high-risk group.

The present study revealed a high prevalence of current SLT use (71.5%) among municipal workers which is significantly higher than the national (21.4%) [39] and state prevalence (29.4%) [40]. Our finding is in line with previous Indian studies reporting a high prevalence of tobacco use in the occupational groups working in construction [38], public transport [39], loom industry [6], mines [41], municipal solid waste management [15], fishermen [42] and daily wagers [8] in a range of 60–91%.

This high prevalence of SLT use in municipal workers can be explained by several reasons. Firstly, a previous nationwide study reported that SLT is associated with the type of physical and mental stress a person experiences during their work schedule [17]. Those who endure much physical effort all or most of the time have highly prevalent SLT use; for example, those who lift heavy loads, work with chemicals, or work with maximum eyesight requirements are the maximum consumers [17]. Municipal workers face significant physical demands in their jobs, including frequent kneeling, stooping, and crouching to collect waste [14]. They are required to lift heavy bags of garbage regularly and work in environments with constant noxious odours [14]. Additionally, they must remain vigilant to protect themselves from the various occupational hazards of waste collection and disposal [14, 15]. Furthermore, owing to long duty hours these workers resort to substance use as a false perception for appetite suppression [43]. A nationwide study of 72,250 individuals reported that the water supply and sewage waste management personnel had one of the highest previous or current use of SLT among the 22 defined occupational groups [17]. 

Secondly, despite the GOI has laid down several tobacco-control efforts such as the integration of brief advice on quitting tobacco in primary care, a national toll-free Quitline, a joint framework for Tuberculosis-tobacco activities, the establishment of Southeast Asia’s first-of-its-kind tobacco testing laboratory, and the mTobacco cessation programme which has 2.1 million self-registered users and ban of gutka sales in 24 states and 3 union territories, enforcement through Cigarette and other Tobacco Products Act (COTPA) [44], the implementation and penetration of these measures remain dismal among populations working in informal sectors and those whose work locations are not fixed or defined, making it difficult to enforce tobacco policies [42–44]. Furthermore, SLT is culturally relevant in our study region and easily accessible due to the unsuccessful ban on gutka [45]. 

Lastly, studies have reported that the SLT ban is often circumvented by distributing gutka in nondescript and twin packaging, which lacks any health hazard warnings or NTQL (National Tobacco Quitline) information [44, 46]. A study reported that 84.6% of tobacco users did not notice the NTQL on the pack [47]. This indicates poor awareness and accessibility for tobacco control measures. Additionally, aggressive uncontrolled advertisement for the promotion and sales of gutka products is rampant in Varanasi [17, 48]. Therefore, both the occupation and accessibility to SLT have contributed to such daunting prevalence of tobacco use in our study settings. Hence, it is recommended that the district-level stakeholders under the National Tobacco Control Programme should identify such high-risk occupational populations and target them with tailored interventions, especially addressing the barriers to quitting tobacco. A comprehensive model which addresses public health interventions in terms of health promotion, prevention, therapeutic and rehabilitative measures should be adopted with proper referral linkages to tackle the problem of tobacco abuse and its outcome among such high-risk populations. Stringent measures to curb advertisements on SLT and to follow the guidelines on labelling of risks of tobacco abuse on the packs should be enforced for cessation of SLT use. Self-help groups may be established and peer education may be taught to discourage tobacco abuse and help workers willing to quit tobacco. Supporting environments to relieve mental and physical stress may be created at the workplace. As the duty hours of these workers constitute a significant part of their lifetime, we recommend a workplace model for tobacco control and prevention (Fig. 3).

We conducted OCS for high-risk participants and observed a screen positivity rate of 32.9% for OPML and OC, significantly higher than the 10.8% rate in the general population of the same district [49]. This highlights the increased risk of OPML in an occupational group compared to the general population, likely due to the higher prevalence of tobacco use—71.5% in our study versus 35.6% in the general population [49]. Furthermore, the higher screen positivity and leukoplakia as the predominant OPML in our study align with the findings of other Indian studies on occupation groups that have conducted OCS [50, 51]. A previous systematic review has reported the malignant transformation (MT) rate of oral leukoplakia as 3.5%, ranging from 0.13–34.0%[52]. Thereby, such a high prevalence of OPML, mainly leukoplakia warrants regular surveillance of the patients for possible malignant transformation, facilitating the early detection of OC.

Furthermore, we observed that lesions such as erythroplakia and those suspicious for cancer were significantly more common in participants with high tobacco dependence, whereas leukoplakia, OSMF, and tobacco pouch keratosis were more frequently observed in those with low to moderate dependence. A systematic review with meta-analysis reported that the malignant transformation rate of erythroplakia is as high as 33.1% (99% CI 13.6%-56.1%), compared to 9.5% (99% CI 5.9%-14.0%) for leukoplakia and 5.2% (99% CI 2.9%-8.0%) for OSMF [5]. Additionally, many epidemiological studies have demonstrated a clear dose-response relationship between tobacco use and the risk of oral cancer or potentially malignant oral diseases [53]. These findings underscore the need for future research to explore the benefits of monitoring tobacco status and conducting regular screenings among high-risk occupational populations, aiming to improve their quality of life, reduce morbidity and mortality, and decrease the financial burden of tobacco-related health issues.

We found that there was a significant decline in nicotine dependence in all active interventions, with maximum impact in the individual behavioral counselling group. Our finding is in line with a systematic review and meta-analysis that reported that behavioral interventions alone showed high efficacy in SLT cessation with positive effects with RR of 0.87 [95% CI 0.7, 1.09] to 3.84 [ 95% CI 2.33, 6.33] [12]. Fiore et al. have reported that among all counselling-related interventions, individual behavioral counselling has the highest quit rates [54]. We selected nicotine dependence as the outcome parameter, as previous studies have reported that the supportive evidence is reasonably strong for nicotine dependence as a key indicator and an important predictor of cessation success [55]. Furthermore, nicotine dependence is the most consistent and significant variable associated with quitting attempts and it can also influence other psychological factors [56]. 

Though we observed reduced nicotine dependence across all intervention groups, we did not observe SLT cessation in any of the intervention groups. Previous Cochrane reviews have shown significantly higher quit rates for smoking in workplace intervention studies. However, evidence of workplace interventions for SLT cessation through systematic reviews is lacking. Several factors may explain the lack of SLT cessation in our study population: (i) previous studies have reported that nicotine levels and, consequently, physical dependence on SLT are significantly higher than for smoking [57–59], (ii) the willingness to quit the SLT is much lower than smoking [60], ( iii) while there is sufficient awareness of the hazards of smoking, knowledge about the risks associated with SLT use is poor among the Indian population, leading to a false perception of SLT as less harmful [61], (iv) unlike smoking, SLT is culturally relevant in our study region, cheaper and easily accessible due to the unsuccessful ban on gutka, and not associated with taboo [17, 48, 62, 63], (v) we conducted baseline interventions followed by measuring the effect of interventions after one year. A meta-analysis has reported that in workplace smoking cessation interventions, after the initial effectiveness, the effect decreased over time and was not present beyond 12 months [64]. Therefore, frequent three to six monthly interval interventions with follow-up visits could have given us a better understanding of the quitting behavior over time, (vi) we did not assess tobacco quit attempts, intentions to quit, and stages of change which could have provided better insights into understanding quitting behaviors in our study population [56], (vii) lastly, many workers cited their working conditions, particularly the obnoxious smell during waste collection and disposal, as a reason for using SLT. Our study did not address these occupational challenges, suggesting that improving working conditions is essential for the program’s success.

### Limitations

Our study faced challenges in terms of limited infrastructure, which hindered the screening process. The absence of washbasins for mouth rinsing impeded proper oral visual examinations. Additionally, inadequate lighting and workstation facilities further compounded these challenges. Despite efforts to invite all tobacco users for follow-up assessments, the follow-up rate was 57.5%. One possible reason for this low rate is that most workers were not available at the main office for screening. Instead, they were working in outreach locations for waste collection, making it difficult for them to come to the main office where the screening facility was arranged. A previous community-based study in similar settings reported that the follow-up rate was just 30%, which can be attributed to several reasons such as poor awareness, misinformation, fear of a cancer diagnosis, perceived fatalism, and poor socio-economic status [44, 49]. Additionally, we did not assess the status of OPML during follow-up which limits our study to assess the effect of cessation interventions on these lesions.

The study collected limited demographic information, mainly age and gender. Important variables such as education level, socio-economic status, marital status, place of residence, co-morbidities, and other substance use habits were not collected. These factors could be potential confounders and significantly influence nicotine dependence, screen positivity, and intervention outcomes. However, it is expected that an occupational group of municipal workers may have similar levels of education and social status. Furthermore, we collected self-reported information on SLT use that was not validated by external sources. Additionally, data on other substance use (e.g., areca nut, smoking and alcohol), which could potentially influence tobacco cessation were not collected. Furthermore, our study specifically targeted high-risk individuals, which restricts the broader applicability of our results to populations with different risk profiles.

### Strength

Our study provides a comprehensive assessment of tobacco cessation interventions, reporting on the burden of tobacco use, and associated OPML and OC observed during screenings. By combining these elements, our study offers a thorough understanding of OC screening outcomes and the effectiveness of tobacco cessation interventions in a high-risk occupational population.

### Conclusion

This study reveals a high prevalence (71.5%) of SLT use among municipal employees in Varanasi, exceeding national and state averages. Contributing factors include the physical demands of their work, cultural acceptance of SLT, and weak enforcement of tobacco control policies. The significant presence of oral potentially malignant lesions (32.9% positivity rate) underscores the need for robust health education, surveillance, and early cancer detection in this high-risk population. While interventions reduced nicotine dependence, complete cessation was not achieved, indicating the need for more intensive strategies. Tailored workplace health models, use of mobile health technologies, and the inclusion of these potential high-risk groups in tobacco control policies are recommended to enhance the effectiveness of SLT cessation efforts. Further research should explore the barriers to quitting SLT in this population.

## Electronic supplementary material

Below is the link to the electronic supplementary material.

Supplementary Material 1

## Data Availability

The datasets generated and/or analyzed to support this study’s findings may be made available from the corresponding author upon reasonable request.
