# Supplementary material for: Prevalence of Oral Potentially Malignant Lesions, Tobacco use, and Effect of Cessation Strategies among Solid Waste Management workers in Northern India: a pre-post intervention study
Source: BMC Oral Health. 2024 Oct 26;24:1292. doi: 10.1186/s12903-024-05087-8 (PMC11515233; doi:10.1186/s12903-024-05087-8)
Supplement: Supplementary file 1 — Supplementary Material 1 [file 12903_2024_5087_MOESM1_ESM.pdf]

Supplementary material

Hindi version of the Fagerstrom Nicotine Dependence Scale-Smokeless tobacco (FTND-ST)

1. How soon after you wake up do you place your first dip?

   (सुबह उठने के कितनी देर बाद आप पहला तम्बाकू सेवन करते है)

☐5 मिनट के भीतर ☐6 - 30 मिनट में ☐31 - 60 मिनट में ☐ 60 मिनट के बाद

2. How often do you intentionally swallow tobacco juice?

   (आप कितनी बार जान-बुझ कर तम्बाकू का रस निगल लेते है)

☐ Always (हमेशा) ☐ Sometimes (कभी कभी) ☐Never (कभी नहीं)

3. Which chew would you hate to give up most?

(आप किस समय के तम्बाकू सेवन को छोड़ना सबसे जयादा नापसन्द करेंगे)

☐The first one in the morning (सुबह का पहले वाला) ☐ Any other (कोई और)

4. How many cans/pouches per week do you use?

(आप एक हफ्ते में कितने डब्बे /थैलियां इस्तमाल करते हैं)

☐More than 3 (तीन से ज्यादा) ☐2 से 3 ☐1

5. Do you chew more frequently during the first hours after awakening than during the rest of the day?

(क्या आप बाकी दिन के मुकाबले में सुबह के पहले तंबाकू सेवन को ज्यादा चबाते है)

☐ Yes (हा) ☐ No (नहीं)

6. Do you chew if you are so ill that you are in bed most of the day?

(जब आप बीमार के कारण बिस्तर पे पड़े हुए है क्या तब भी आप तंबाकू सेवन करते हैं?)

☐ Yes (हा) ☐No (नहीं)
